# A Novel Foodstuff Mixture Improves the Gut–Liver Axis in MASLD Mice and the Gut Microbiota in Overweight/Obese Patients

**DOI:** 10.3390/antiox13060664

**Published:** 2024-05-29

**Authors:** Rebeca Rosas-Campos, Ana Soledad Sandoval-Rodríguez, Jonathan Samael Rodríguez-Sanabria, Ángel Omar Vazquéz-Esqueda, Carlos Roberto Alfaro-Martinez, Rebeca Escutia-Gutiérrez, Natali Vega-Magaña, Marcela Peña-Rodríguez, José Sergio Zepeda-Nuño, Mauricio Andrade-Marcial, Yolanda Campos-Uscanga, Luis Felipe Jave-Suárez, Arturo Santos, Eira Cerda-Reyes, Mónica Almeida-López, Erika Martínez-López, Luis Alonso Herrera, Juan Armendariz-Borunda

**Affiliations:** 1 Institute for Molecular Biology in Medicine and Gene Therapy, Department of Molecular Biology and Genomics, Health Sciences University Center, University of Guadalajara, Guadalajara 44340, Mexico; rosas.rcam@gmail.com (R.R.-C.); soledad.sandoval@academicos.udg.mx (A.S.S.-R.); samael.rodsan@gmail.com (J.S.R.-S.); aovecf@gmail.com (Á.O.V.-E.); carlos.alfaro2286@alumnos.udg.mx (C.R.A.-M.); rebeca.escutia@academicos.udg.mx (R.E.-G.); 2 Departamento Académico de Ciencias Básicas, Universidad Autónoma de Guadalajara, Zapopan 45129, Mexico; 3 Instituto de Investigación en Ciencias Biomédicas (IICB), Health Sciences University Center, University of Guadalajara, Guadalajara 44340, Mexico; alejandra.vega@academicos.udg.mx; 4 Laboratorio de Diagnóstico de Enfermedades Emergentes y Reemergentes (LaDEER), Health Sciences University Center, University of Guadalajara, Guadalajara 44340, Mexico; marcela.pena@cucs.udg.mx; 5 Centro de Investigación y Diagnóstico de Patología, Health Sciences University Center, University of Guadalajara, Guadalajara 44340, Mexico; jsergio.zepeda@academicos.udg.mx; 6 Unidad de Biotecnología, Centro de Investigación Científica de Yucatán, Merida 97205, Mexico; mauricioandrademarcial@gmail.com; 7 Instituto de Salud Pública, Universidad Veracruzana, Xalapa 91190, Mexico; ycampos@uv.mx; 8 División de Inmunología, Centro de Investigación Biomédica de Occidente, Instituto Mexicano del Seguro Social, Guadalajara 44340, Mexico; lfjave@gmail.com; 9 Tecnologico de Monterrey, School of Medicine and Health Sciences, Zapopan 45138, Mexico; arturo.santos@tec.mx (A.S.); lherreram@tec.mx (L.A.H.); 10 Hospital Central Militar, Mexico City 11200, Mexico; arieirace@yahoo.com.mx; 11 Health Sciences University Center, University of Guadalajara, Guadalajara 44340, Mexico; moniconia@gmail.com; 12 Institute of Translational Nutrigenetics and Nutrigenomics, Department of Molecular Biology and Genomics, Health Sciences University Center, University of Guadalajara, Guadalajara 44340, Mexico; erika.martinez@academicos.udg.mx; 13 Cancer Research Unit, National Institute of Cancerology-Institute of Biomedical Research, National Autonomous University of Mexico (UNAM), Mexico City 70228, Mexico

**Keywords:** *Opuntia ficus indica*, *Theobroma cacao*, *Acheta domesticus*, MASLD, Mexican diet, obesity

## Abstract

Microbial community control is crucial for maintaining homeostasis of the gut–liver axis in metabolic dysfunction-associated steatotic liver disease (MASLD). Here, we show that supplementation with a mixture of Mexican foodstuffs (MexMix)—*Opuntia ficus indica* (nopal), *Theobroma cacao* (cocoa) and *Acheta domesticus* (crickets)—enriches several beneficial taxa in MASLD mice and overweight/obese humans. Thus, MexMix induces an important prebiotic effect. In mice, a restoration of intestinal health was observed due to the increased short-chain fatty acids (SCFAs) and intestinal crypt depth, *Ocln* and *Cldn1* expression, and decreased *Il6* and *Tnfa* expression. MexMix significantly reduced steatosis in the mice’s liver and modified the expression of 1668 genes. By PCR, we corroborated a *Tnfa* and *Pparg* decrease, and a *Cat* and *Sod* increase. In addition, MexMix increased the hepatic NRF2 nuclear translocation and miRNA-34a, miRNA-103, and miRNA-33 decline. In overweight/obese humans, MexMix improved the body image satisfaction and reduced the fat intake. These findings indicate that this new food formulation has potential as a therapeutic approach to treat conditions associated with excessive consumption of fats and sugars.

## 1. Introduction

Metabolic dysfunction-associated steatotic liver disease (MASLD) represents the contemporary nomenclature for steatotic liver disease linked to metabolic syndrome [1]. MASLD affects half of overweight/obese adults, becoming the most common liver disease worldwide, exerting a significant impact on liver-related morbidity and mortality [2]. As a consequence, it represents an economic burden for health systems. 

The prehispanic diet was based on around 200 different foods, including corn, black beans, nopal, chili, pumpkin, chia seeds, turkey, fish, and insects such as grasshoppers, crickets, and agave worms. Due to this, obesity was practically absent from the pre-Columbian population [3]. Nowadays, lifestyle has dramatically affected diet, where occidental societies have reduced the variety of foods consumed, decreasing fiber and antioxidants, and increasing fat and sugars, causing an increase in obesity and related comorbidities, such as MASLD [4]. Since no approved drug therapy is available for MASLD, lifestyle modifications remain the main treatment, though adherence to this treatment is poor [5]. This situation calls for urgent attention to develop new therapeutic strategies on a large scale. 

The aim of this study was to evaluate the ingestion of specific ancestral Mexican foods to ameliorate MASLD by means of a mixture of *Opuntia ficus indica*, *Theobroma cacao* and *Acheta domesticus*. This mixture of foods was called MexMix for short. Our idea stems from the ecological benefit of using crickets as a protein source over traditional livestock like cattle.

Previously, our group demonstrated that MexMix improved the obesogenic features, through restoring the body weight, triglycerides, insulin, and leptin levels, in a murine model of mild obesity (35% Kcal from fat in the diet) [6]. However, the potential effects of MexMix on more severe diet induced-MASLD (60% Kcal from fat) are still unknown. Furthermore, the molecular and epigenetic mechanisms by which MexMix carries out its effects need to be deeply understood. In addition, it is essential to test the effect and safety of MexMix in patients to determine its potential as an adjuvant in metabolic diseases.

## 2. Materials and Methods

### 2.1. Animals and Diet

Four-week-old male C57BL/6J mice (weighing 20–25 g) were housed in a 12 h light–dark cycle at 22 °C ± 1 and received care in accordance with the Official Mexican Norm NOM-062-ZOO-1999. The University of Guadalajara’s Ethics Committee approved the animal study (ID: CI-09420). After one week of acclimation, the mice were randomized into three groups: the ND group received a standard diet for 18 weeks (Envigo T.2018S.15) (*n* = 8); the HF/FS group was fed with a high-fat diet (60% Kcal in fat) plus a carbohydrate-rich drink (2.31% fructose, 1.89% sucrose) for 18 weeks (*n* = 8); and the MexMix group received a HF/FS diet up to week ten and then switched for eight additional weeks to a HF/FS diet supplemented with a Mexican foods mixture: nopal (6.7%), cocoa (8.7%) and crickets (8.7%) (*n* = 8) (Table S1). The food and drink intake was measured three times a week at 9:00 a.m. and the energy intake was calculated. Finally, 48 h prior to sacrifice, the mice were fasted for 4 h to perform an insulin tolerance test (ITT). Human recombinant short-acting insulin was administered intraperitoneally at a dose of 0.75 U/kg. 

After 4 h of fasting, the animals were sacrificed under anesthesia. Epididymal and visceral fat, colon and liver tissue were immediately collected, weighed, and fixed with 10% paraformaldehyde for histological examination or stored at −80 °C until analysis. Serum was obtained from blood drained from the ocular vein and fecal samples from the colon were collected, and both samples were stored at −80 °C until analysis. The triglycerides and cholesterol serum levels were measured in a VITROS^TM^ 350 analyzer (Ortho Clinical Diagnostics, Raritan, NJ, USA). The concentrations of ghrelin, GIP, GLP-1, glucagon, insulin, leptin, PAI-1, and resistin were measured using a Bio-Plex Pro™ mouse diabetes 8-plex immunoassay (Bio-Rad, Hercules, CA, USA), and the levels of adiponectin with a Bio-Plex Pro™ mouse adiponectin 8-plex immunoassay (Bio-Rad, Hercules, CA, USA), following the manufacturer’s instructions. 

### 2.2. Histological Analysis and Immunohistochemistry

Hematoxylin and eosin (H&E) staining was performed in 5 μm thick sections of epididymal adipose, liver lobules, and colon tissues. The liver was analyzed for hepatocyte ballooning, inflammatory nodules, microsteatosis and macrosteatosis. The percentage of steatosis was quantified using ImagePRO software version 6.3. In adipose tissue, the cell architecture, inflammatory infiltrate, and adipocyte size (µm^2^) were analyzed using ImagePRO. In colon sections, the crypt size (µm) was measured with ImagePRO. Histological analysis was performed by two pathologists blinded in at least 10 microscopic fields/mice at 20× magnification. 

For immunohistochemistry, the Claudin-1 and CD45 protein expression was determined in the colon. Sections (3 μm) were processed using the Novolink polymer detection system (Leica Biosystems, Newcastle, Ltd., Newcastle upon Tyne, UK) following the manufacturer’s guidelines. Antigen retrieval was carried out using EDTA buffer during 50 min for Claudin-1 and 40 min for CD45. Subsequently, the sections were subjected to an overnight antibody incubation at 4 °C at a dilution of 1:250 for Claudin-1 and 1:800 for CD45 (Table S2). Aperio LV1 IVD equipment and Aperio Imagescope software v.12.4.6.5003 were employed to document the protein expressions. 

### 2.3. 16S rRNA Amplicon Sequencing and Microbiota Diversity Analysis

The total DNA was extracted from the colon fecal samples using the QIAamp Fast DNA Stool Mini Kit according to the manufacturer’s protocol. The DNA concentration was measured using the Qubit^®^ dsDNA HS kit; the purity and integrity were analyzed in 1% agarose gels. 

The Illumina MiSeq System (Illumina, San Diego, CA, USA) protocol was followed for the library preparation for 16S metagenomic sequencing. Primers for the V3 and V4 regions (Table S3) were amplified with Platinum Taq DNA Polymerase High Fidelity (Invitrogen, Waltham, MA, USA). The PCR amplicon was purified using AMPure XP^®^ magnetic beads (Beckman Coulter, Indianapolis, IN, USA) and quantified in Qubit^®^ equipment according to the product indications. Index incorporation was achieved with the Nextera XT Index Kit v2 Set A (No. Cat. FC-131-2001, Illumina, San Diego, CA, USA) by a second PCR. Lastly, the amplicons were pooled to equimolar concentrations and placed into a solution tube with a concentration of 4 nmol/L. The library was then denatured and MiSeq Sample Loading (MiSeq Reagent V3 600-cycle kit, Illumina, San Diego, CA, USA) was carried out following the provider’s protocol.

The demultiplexed sequencing data were analyzed using QIIME2 v2021.8 [7]. Using the DADA2 plugin, the sequences were denoised, filtered and trimmed to 220 nt length according to standard quality-filtering procedures. The amplicon sequence variants (ASVs) were classified against the Silva_138 database. Beta diversity metrics, UniFrac analysis-weighted and -unweighted, and permutational multivariate analysis of variance (PERMANOVA) were tested. A three-dimensional scatter plot was generated using Principal Coordinate Analysis (PCoA) in MicrobiomeAnalyst. The Kruskal–Wallis test was used to determine statistical differences in the alpha diversity. 

To identify differentially abundant bacterial taxa, linear discriminant analysis effect size (LEfSe) analysis was performed using the Galaxy workflow (https://huttenhower.sph.harvard.edu/galaxy/root, accessed on 9 January 2023). 

PICRUSt2 was performed to predict the functional pathways and a Kyoto Encyclopedia of Genes and Genomes (KEGG) Orthology (KO) abundance table was generated [8]. Conversion of the KO abundance tables into KEGG pathway abundance tables identified statistical differences in the pathways between HF/FS and MexMix that were plotted using ggpicrust2 [9]. 

Lastly, a heatmap was created in R (v4.2.2) with Spearman correlation analysis using bacterial taxa at the genera level and variables of interest (microbiome package, v1.17.41). 

### 2.4. Analysis of Fecal Short-Chain Fatty Acids (SCFAs)

Three SCFAs were measured (acetate, propionate, and butyrate). A total of 20–30 mg of feces was shaken with 200 µL of water until homogeneous. Then, 40 µL HCl 0.1 M, 20 mg citric acid, and 40 mg of NaCl were added and mixed. Next, 200 µL of the compound reagent solution (N-butanol, tetrahydrofuran and acetonitrile in a 50:30:20 ratio) was added and vortexed for 1 min. The sample was centrifuged at room temperature for 10 min at 13,000× *g*. The supernatant was filtered through a Whatman GD/X syringe PVDF membrane filter 0.22 µm pore (MERCK, Billerica, MA, USA) and analyzed in the Shimadzu GC 2010 gas chromatograph with flame ionization detector (FID) (Shimadzu Scientific Instruments, Kyoto, Japan). To perform the analysis, a Mega-Acid^®^ high-polarity stationary-phase column (MEGA, Legnano, MI, Italy) was used. The data were analyzed with LabSolutions-Chromatography Data System software (Shimadzu Scientific Instruments, Kyoto, Japan). The quantification of the SCFAs was calculated by interpolation with standard curves.

### 2.5. Microarrays

The total RNA was extracted from 20 mg of liver using TRIzol reagent (Invitrogen in Carlsbad, CA, USA). Employing agarose gel electrophoresis, the 28S and 18S rRNA ratio was analyzed, and a NanoDrop ND-2000 spectrophotometer (Thermo Fisher Scientific, Waltham, MA, USA) was used for the quantification. The total RNA was pooled from five mice per group. The 23,232 genes were hybridized for the *Mus musculus* genome using double-channel microarrays following the Microarray Facility UNAM guidelines (http://microarrays.ifc.unam.mx, accessed on 22 May 2022). Image quantification was performed using genArise Microarray Analysis v2.0 and adjusted *p* values of 0.05 and Z score values of >−1.5 and <1.5 were considered significant. 

Gene Ontology was used for fold enrichment analysis of the biological processes with genes with a Z score value of >1.5 employing ShinyGO v0.76.3 (http://bioinformatics.sdstate.edu/go/, accessed on 23 September 2022). Finally, heatmaps were constructed using the ComplexHeatmap package in R (v4.2.2). 

### 2.6. RT-qPCR

The total RNA was isolated from the liver and colon. To synthesize cDNA, 2 μg of total RNA and M-MLV reverse transcriptase (Invitrogen, Carlsbad, CA, USA) was used. The cDNA was subject to qPCR procedures using 2 μL and TaqMan probes (Table S4) according to the manufacturer’s instructions. All the samples were run in triplicate, normalized using GAPDH as a housekeeping gene, and analyzed using the ΔΔCt method. 

### 2.7. miRNA Extraction and Expression

The liver miRNAs were extracted utilizing the miRVana miRNA isolation kit (Thermo Fisher Scientific, Waltham, MA, USA) following the manufacturer’s protocol. Ten nanograms of miRNAs were subjected to retrotranscription using the Advanced miRNA cDNA synthesis kit (Thermo Fisher Scientific, Waltham, MA, USA), again according to the manufacturer’s instructions. The cDNA was subjected to a 1:10 dilution and 2.5 µL was utilized for the qPCR reactions using TaqMan probes, targeting mmu-miR-34a-5p, mmu-miR-122-5p, mmu-miR-33, and mmu-miR-103-3p (Table S4). Gene expression normalization was performed utilizing mmu-miR-16-5p and analyzed using the ΔΔCt method.

### 2.8. Immunoblotting

The nuclear fraction proteins were extracted from liver tissue. The proteins were separated using SDS-PAGE gel, transferred to PVDF membranes, and immunoblotted overnight at 4 °C with specific antibodies (NRF2, H3K14 and lamin B1; 1:1000; Table S2). The membrane was revealed with anti-Mouse-immunoglobulin G(IgG)-peroxidase (POD)/anti-Rabbit-IgG-POD (1:10,000). The band intensities were detected with ChemiDoc XRS+ and quantified with ImageJ software version 1.53. Lamin B1 was used as the loading control.

### 2.9. Participants Selection and Study Design

A pilot study with a pre-post-intervention comparison was designed to evaluate MexMix supplementation in humans. Participants were recruited by social media posts. The inclusion criteria were adults aged 18–40 years, body mass index (BMI) > 25.0 kg/m^2^, no recent antibiotics (60 days), defecation frequency of at least once every 3 days and a maximum of twice a day, and an intake <25 g fiber/day. The 24 h dietary recall was performed to capture the food intake on two weekdays and one weekend day. A dietary test was applied pre-treatment and at the study’s end, and the calorie and fiber intake were evaluated.

Thirty-three participants were recruited, but only twenty were eligible. Moreover, 4 of the 20 were excluded due to intervention. All the participants signed informed consent, understanding the study goals/methods. The University of Guadalajara’s Ethics Committee approved this study (ID: CI-01421).

### 2.10. Dietary Intervention

MexMix consists of a mixture of 10 g per each food: dehydrated nopal, cocoa powder and dehydrated cricket, packed in a wrapping (Table S5). MexMix was incorporated into breakfast and added with 330 mL of liquid. The intervention spanned six weeks, during which the participants maintained their regular lifestyles and eating habits. To ensure a real-life impact, the participants were monitored biweekly by a nutritionist to track any dietary/physical activity changes and gastrointestinal symptoms (diarrhea, constipation, etc.) or any other adverse effects. Compliance was validated through returned packaging and daily reminders via phone messages.

### 2.11. Anthropometry, Recollection Feces and Serum Samples

The participants’ body weight, BMI, visceral fat area (VFA), muscle mass and fat mass was measured by bioimpedance (InBody 720, Biospace) in the morning after 12 h of fasting. 

Blood and stool samples were collected after 12 h of fasting. Cholesterol, triglycerides, HDL, LDL, VLDL, AST and ALT were measured in a VITROS^TM^ 350 analyzer (Ortho Clinical Diagnostics, Raritan, NJ, USA). Microbiota diversity analysis was performance from 200 mg of fresh feces and as previously described (Section 2.3). 

### 2.12. Psychological Test

Psychological well-being (PWB) was determined using the Ryff Psychological Well-being Scale, previously validated in the Mexican population [10]. To evaluate body image satisfaction, the Body Shape Questionnaire (BSQ)-18 scale was used, previously validated in Mexican university students [11].

### 2.13. Statistical Analysis

Data are presented as the mean ± SD or mean ± SEM for graphical representations. For the murine model variables, normality was assessed using the Shapiro–Wilk test. For variables that exhibited a normal distribution, statistical significance was evaluated using a parametric one-way analysis of variance (ANOVA) followed by Tukey’s post hoc test. For variables that did not follow a normal distribution, significance was determined using the non-parametric Kruskal–Wallis test. For the pilot study variables, the Kolmogorov–Smirnov test was used to establish the normality of the variables, and to analyze differences between baseline and after 6 weeks, paired *t*-test was used for variables with a normal distribution and Wilcoxon matched-pair for variables that did not follow a normal distribution. The data analysis was performed in the R environment (v4.2.2) and graphs were generated using GraphPad Prism 8.0. A significance level of *p* < 0.05 was considered statistically significant. 

## 3. Results

### 3.1. MexMix Induced Metabolic and Adipogenic Variations in Mice

**Figure 1:**
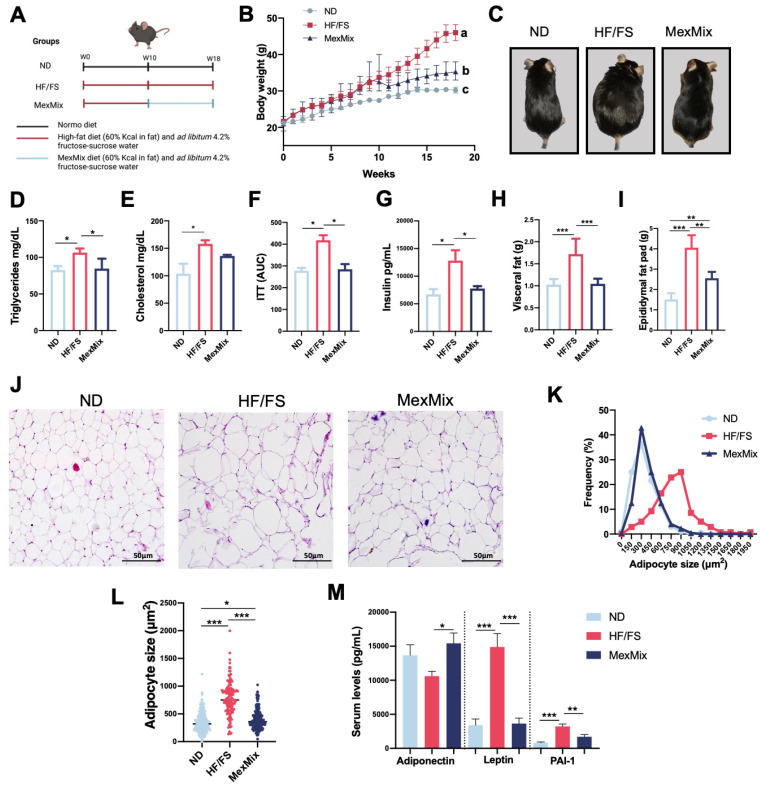
MexMix induced metabolic and adipogenic variations in mice. (**A**) Mice model diagram. (**B**) Body weight gain. (**C**) Representative mice photo per group. (**D**) Triglycerides levels. (**E**) Cholesterol levels. (**F**) Total area under the curve calculated from ITT data. (**G**) Insulin levels. (**H**) Visceral fat weight. (**I**) Epididymal fat pad weight. (**J**) Hematoxylin and eosin staining of epididymal fat. Photos at ×20 magnification. (**K**) Adipocyte size distribution. (**L**) Adipocyte area. (**M**) Adiponectin, leptin and PAI-1 serum levels. Data represent mean ± SEM (* *p* < 0.05; ** *p* < 0.01; *** *p* < 0.001). a, b, c: Statistical differences between groups.

First, we analyzed the effects of MexMix supplementation in mice (Figure 1A). The MexMix group showed a significant increase in Kcal consumption during the supplementation period (Table S6), but even so, these mice displayed a reduced body weight (24%) compared to the HF/FS animals (Figure 1B). In Figure 1C, a representative picture of an animal per group is presented, where the HF/FS-mice show clear obesity, whereas the MexMix mice’s body mass is similar to the ND mice. In addition, supplementation with MexMix exhibited a significant decrease in the triglycerides levels (Figure 1D) and an evident tendency toward cholesterol level reduction (Figure 1E). MexMix supplementation prevented the development of insulin resistance (Figure 1F) and accordingly caused a significant reduction in insulin levels (Figure 1G). 

A decrease in visceral and epididymal fat was observed in the MexMix animals, where values are like those in the ND group (Figure 1H,I). At the same time, the MexMix diet restored the shape and size of the adipocytes, and the histological cell features looked similar to ND adipocytes (Figure 1J). In the HF/FS animals, around 45% of the adipocytes had an area >900 μm^2^, while in the MexMix group, only 2.6% of cells had a surface area >900 μm^2^ (Figure 1K). Therefore, the MexMix adipocyte mean area significantly decreased 1.95-fold compared to the HF/FS adipocytes (Figure 1L). 

An increase of adipocyte size is associated with a deregulation in metabolic-related hormone secretion [12]. In our results, MexMix significantly restored the secretion of MASLD development-related adipokines. Leptin showed a significant 38% reduction in the MexMix group, while Plasminogen Activator Inhibitor-1 (PAI-1) also significantly decreased (Figure 1M). Remarkably, adiponectin, an anti-inflammatory adipokine and inducer of β-oxidation in liver [13], was significantly increased in the MexMix group, even more than in the ND group (Figure 1M). No differences were found in the serum levels of ghrelin, GIP, GLP-1, glucagon, and resistin (Figure S1). 

### 3.2. MexMix Changed Gut Microbiota Composition in Mice

**Figure 2:**
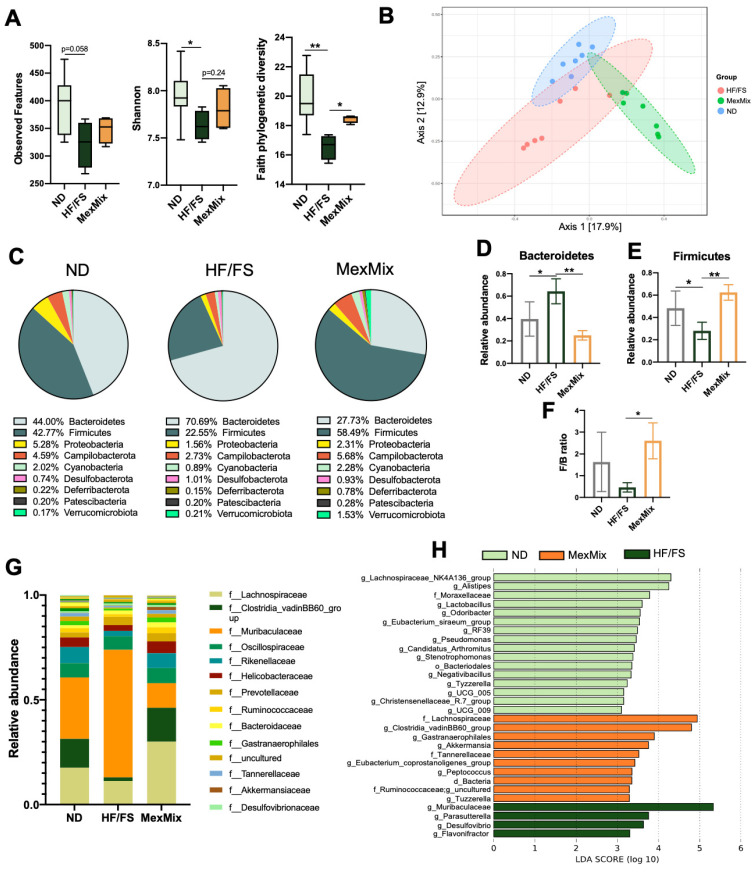
MexMix changed gut microbiota composition in mice. (**A**) Alpha diversity: observed features, Shannon’s index, and Faith phylogenetic diversity. (**B**) Scatter plot generated using PCoA to analyze β-diversity. (**C**) Pie chart of most abundant bacterial phyla (>0.1%) and their relative abundance. (**D**) Bacteroidetes relative abundance. (**E**) Firmicutes relative abundance. (**F**) Firmicutes/Bacteroidetes ratio. (**G**) Relative abundance of most abundant bacterial families. (**H**) LEfSe analysis with bacterial taxa differentially enriched in each group. Threshold on logarithmic LDA score was set to 2.0. Data represent mean ± SEM (* *p* < 0.05; ** *p* < 0.01). The box and whisker plots represent the minimum value, first quartile, median, third quartile, and maximum value.

We characterized the gut microbiota using 16rRNA sequencing (Table S7). We found that MexMix did not modify the observed features but tended to increase the Shannon diversity and significantly increased the Faith phylogenetic diversity (Figure 2A). We demonstrated that MexMix supplementation had a strong impact on community composition since aggrupation by PCoA clearly separated the ordination of MexMix from the HF/FS and ND groups. This separation was statistically significant according to the PERMANOVA test (MexMix vs. ND *p* = 0.003; MexMix vs. HF/FS *p* = 0.03) (Figure 2B). This difference might be related to the significant decrease in the relative profusion of Bacteroidetes (Figure 2C,D) and increased Firmicutes abundance (Figure 2C,E), and consequently, the Firmicutes/Bacteroidetes ratio was also increased (Figure 2F). Furthermore, we found a significant increase in Cyanobacteria and Verrucomicrobiota phyla in MexMix (Figure 2C and Table S8).

At the family level, MexMix induced an increase in the relative abundance of the Lachnospiraceae, Ruminococcaceae and Akkermansiaceae families (Figure 2G). LEfSe analysis revealed that 10 bacterial taxa characterized the MexMix group, including Lachnospiraceae, *Clostridia_vadinBB60_group*, *Gastranaerophilales*, *Akkermansia*, Ruminococcaceae and *Eubacterium_coprostanoligenes_group*. Noteworthily, most of them produce SCFAs (Figure 2H). On the other hand, four disease-associated bacterial taxa characterized the HF/FS group (Muribaculaceae, *Parasutterella*, *Desulfovibrio* and *Flavonifractor*) (Figure 2H).

**Figure 3:**
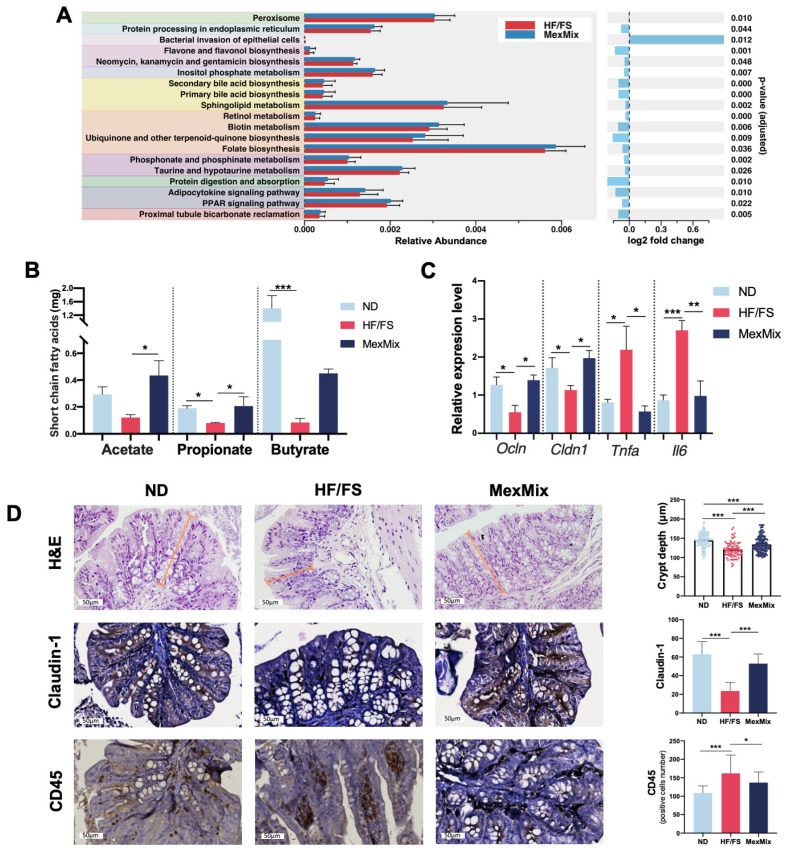
MexMix intervention restored intestinal health in mice. (**A**) Representative pathways of PICRUSt2 functional prediction analysis of the MexMix group compared to HF/FS. A fold-change oriented to the right indicates an upregulation of the pathway in the HF/FS group, whereas a fold-change oriented to left indicates an upregulation in the MexMix group. (**B**) Short-chain fatty acids concentration in colon feces. (**C**) Colon expression at mRNA level of *Ocln*, *Cldn1*, *Tnfa* and *Il6*. (**D**) Microscopic photographs of colon sections in H&E staining and crypt depth. Immunohistochemistry of Claudin-1 and CD45. Brown color represents specific protein immunostaining, and light violet color indicates hematoxylin staining. Photos at ×20 magnification. Data represent mean ± SEM (* *p* < 0.05; ** *p* < 0.01; *** *p* < 0.001).

To predict the functional pathways, PICRUSt2 analysis was performed. Differential abundance analysis identified 45 categories in the KEGG that significantly differed between the MexMix and HF/FS groups (Table S9). Representative pathways are shown in Figure 3A, where the “Protein digestion and absorption” and “Flavone and flavonol biosynthesis” pathways linked to nutrients contained in MexMix are significantly increased, while “Bacterial invasion of epithelial cells” was decreased. 

### 3.3. MexMix Intervention Restored Intestinal Health in Mice

In agreement with the enrichment in SFCA-producing bacteria, we found that MexMix significantly increased the fecal acetate and propionate concentrations and clearly leaned toward an increased butyrate amount (Figure 3B). The SCFAs, a product of fiber fermentation by the gut microbiota, act as a source of energy and are associated with gut barrier improvement [14]. Moreover, MexMix induced the upregulation of tight junction protein gene expression, specifically *Ocln* and *Cldn1* (Figure 3C), and a significant increase in the crypt depth (Figure 3D). Immunohistochemistry in colon sections corroborated the increase in Claudin-1 in the MexMix-mice, which led to a decrease in inflammatory infiltrates in the lamina propria expressed by a reduction in CD45+ cells after supplementation with MexMix (Figure 3D). These findings match with a substantial decline in *Il6* and *Tnfa* gene expression by MexMix (Figure 3C). 

Interestingly, we found statistically significant correlations between the bacterial taxa that characterized MexMix supplementation and several studied variables (Figure S2). Lachnospiraceae and Ruminococcaceae negatively correlated with *Il6* expression and *Akkermansia* positively correlated with adiponectin.

### 3.4. MexMix Reduced Liver Steatosis and Modified Hepatic Total Gene Expression in Mice

**Figure 4:**
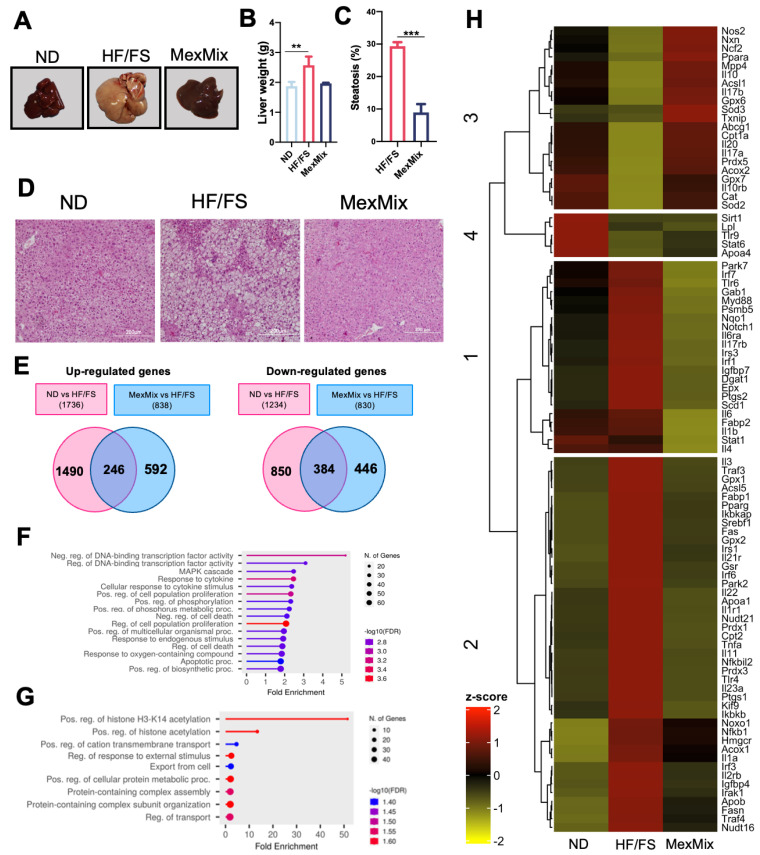
MexMix reduced liver steatosis and modified hepatic total gene expression in mice. (**A**) Representative liver images from each group. (**B**) Liver weight. (**C**) Percent of liver steatosis. (**D**) Microscopic photographs of H&E staining of liver sections. Photos at ×20 magnification. (**E**) Venn diagrams of total upregulated and downregulated genes with Z score < 1.5 and >−1.5, respectively. (**F**) Biological processes significantly enriched in the HF/FS group according to Gene Ontology (GO) database. (**G**) Biological processes significantly enriched with MexMix supplementation. (**H**) Heatmap representing hepatic expression profiles of genes involved in inflammatory processes, oxidative stress and lipid metabolism. Red bars indicated genes upregulated with a Z score up to 1.5 and downregulated genes with a Z score down to −1.5 are represented with green bars. Clusters generated according to the gene expression level are indicated with bars and numbers on left side. Data represent mean ± SEM (** *p* < 0.01 and *** *p* < 0.001).

Liver lipid accumulation is the main characteristic of MASLD. Figure 4A shows representative pictures of livers; the HF/FS livers had the biggest size and a light yellowish color due to fat accumulation, while the MexMix livers had a size and color like the ND livers. Consequently, the liver weight strongly tended to decrease in the MexMix animals (Figure 4B). The MexMix animals showed a histology like the ND group (Figure 4D) and a significant reduction in the steatosis percentage (Figure 4C).

As depicted in Figure 4E, a total of 838 genes were upregulated in MexMix vs. HF/FS and 830 were downregulated (Figure 4E). Sixteen biological processes were significantly enriched in the HF/FS group, including “MAPK cascade” and “response to cytokine”, among others (Figure 4F). MexMix enriched nine biological processes, remarkably “positive regulation of histone H3-K14 acetylation”, which was the process with the highest fold enrichment (Figure 4G).

To analyze the expression of genes involved in inflammatory processes, oxidative stress, and lipid metabolism, a heatmap was built. In Figure 4H, a similar color and shape between the ND and MexMix groups are displayed for most genes. Outstandingly, in the MexMix group, the expression of genes involved in lipid and glucose metabolism showed an increase in *Ppara*, *Cpt1a*, and *Acox2*, and a decrease in *Pparg*, *Srebf1*, *Fabp2*, *Notch1*, *Irs3*, *Scd1*, *Fas*, *Igfbp7*, and *Fasn*. Furthermore, in oxidative stress-associated genes, MexMix supplementation increased the expression of *Sod3*, *Cat*, *Sod2*, *Txnip*, *Prdx5*, *Nos2*, *Gpx8*, *Gpx7*, and *Ncf2*. Regarding inflammatory processes, the MexMix group had an increase in gene expression of *Il10rb*, *Il10*, *Il17b*, *Il17a* and *Il20*, while the expression of *Tlr4*, *Tlr6*, *Myd88*, *Il6ra*, *Irf1*, *Il22*, *Irf7*, *Il6*, *Il1b*, *Stat1* and *Il4* was downregulated (Figure 4H).

### 3.5. MexMix Modified Protein and Gene Expression and Epigenetic Markers

**Figure 5:**
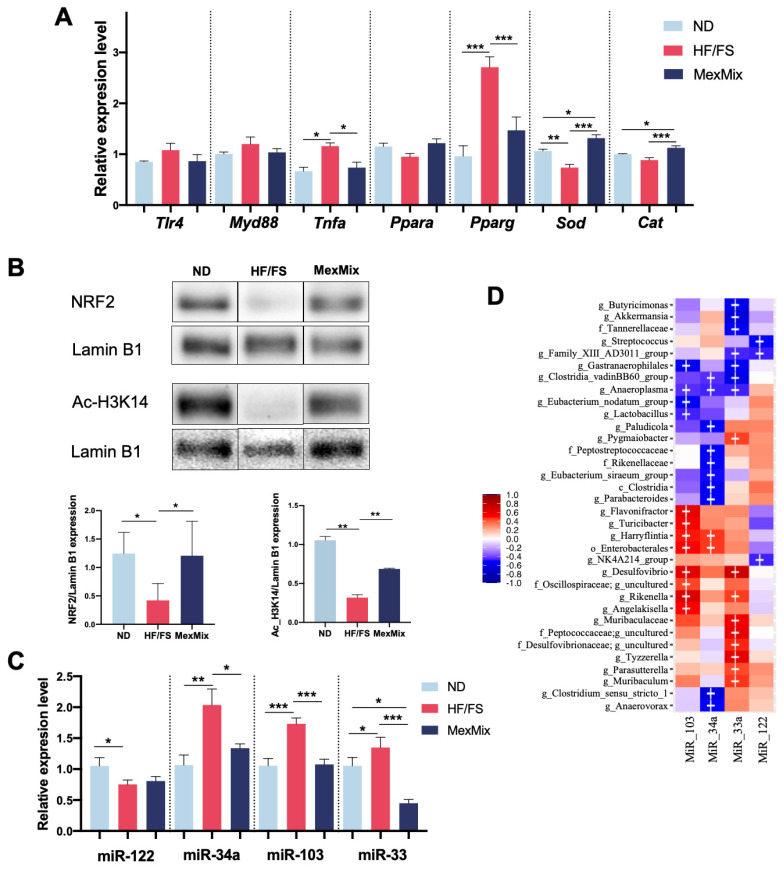
MexMix modified protein and gene expression and epigenetic markers. (**A**) Hepatic expression at mRNA level of *Tlr4*, *MyD88*, *Tnfa*, *Ppara*, *Pparg*, *Sod* and *Cat*. (**B**) Nuclear protein expression of NRF2 and H3K14 in mice liver. (**C**) Hepatic expression of selected miRNAs involved in MAFLD pathogenesis. (**D**) Heatmap of the Spearman correlations of miRNAs expression and bacterial abundance. Red shades indicate positive correlations, while blue shades indicate negative correlations. Statistically significant correlations (*p* < 0.05) are denoted by (+). Data represent mean ± SEM (* *p* < 0.05; ** *p* < 0.01; *** *p* < 0.001).

Next, we selected some genes from each biological process to corroborate the changes in expression. First, we hypothesized that the enrichment of the “response to stimuli by cytokines” and “MAPKs cascade” pathways (Figure 4F) could be due to bacteria translocation and its recognition via the TLR4/Myd88 pathway. However, we did not find differences in the hepatic mRNA levels of TLR4 and Myd88 (Figure 5A). Noteworthily, supplementation with MexMix considerably decreased *Tnfa* expression. As to genes involved in lipid metabolism, a decrease in *Pparg* expression was corroborated. Additionally, the MexMix animals showed a significant increase in *Sod* and *Cat* expression—important antioxidant enzymes—even greater than in the ND group (Figure 5A). These facts could be related to an increase in the nuclear translocation of nuclear factor erythroid 2-related factor (NRF2) by MexMix (Figure 5B). Lastly, MexMix increased the acetylation of H3K14, as confirmed by WB (Figure 5B). 

As previously known, gene expression is also regulated by miRNAs, and based on this, we analyzed the MexMix supplementation effect on these epigenetic regulators implicated in MASLD development. The MexMix group presented a significant decrease in the expression of miR-34a, miR-103, and miR-33; however, no differences were found in miR-122 by MexMix (Figure 5C). It has been proposed that the intestinal microbiota could modulate hepatic miRNAs expression [15]. Notably, we identified statistically significant correlations between the bacterial taxa associated with MexMix supplementation and the four miRNAs (Figure 5D).

### 3.6. MexMix Pilot Study in Overweight/Obese Participants

**Figure 6:**
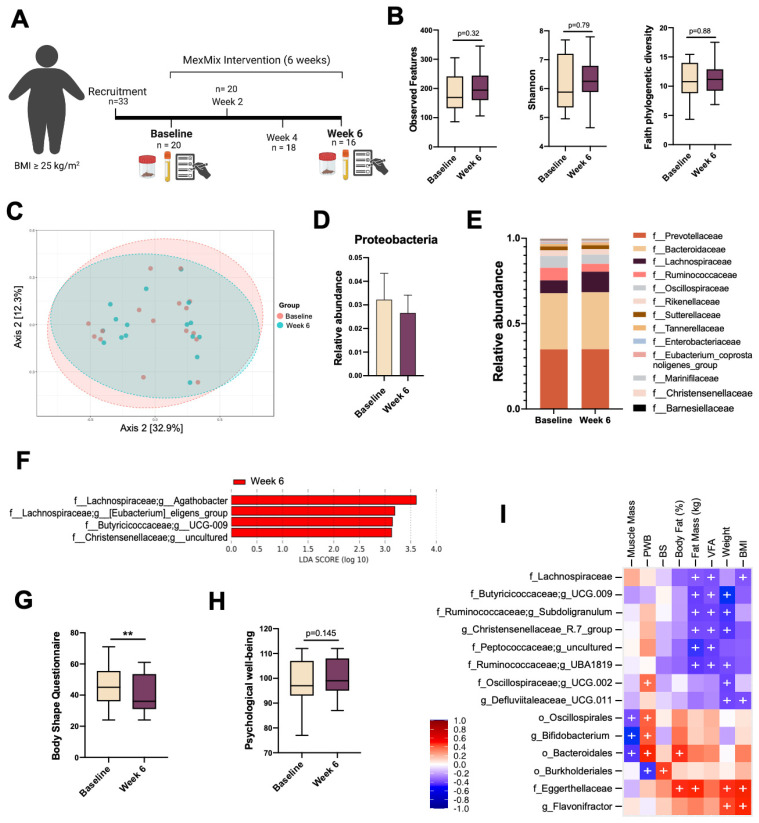
MexMix pilot study in overweight/obese participants. (**A**) Diagram of MexMix intervention in participants. (**B**) Alpha diversity: observed features, Shannon’s index and Faith phylogenetic diversity. (**C**) Scatter plot to analyze β-diversity generated using PCoA. (**D**) Proteobacteria relative abundance. (**E**) Relative abundance of most abundant bacterial families. (**F**) LEfSe analysis with bacterial taxa differentially enriched after MexMix intervention. Threshold on logarithmic LDA score was set to 2.0. (**G**) Body dissatisfaction. (**H**) Psychological well-being. (**I**) Heatmap of the Spearman correlations of clinical metadata and bacterial abundance. Red shades indicate positive correlations, while blue shades indicate negative correlations. Statistically significant correlations (*p* < 0.05) are denoted by (+). Data represent mean ± SEM (** *p* < 0.01). The box and whisker plots represent the minimum value, first quartile, median, third quartile, and maximum value.

Since the results in mice implied advantageous effects, we decided to test MexMix in a clinical scenario (Figure 6A). Despite the fact that MexMix contributes to caloric intake (119 Kcal), the participants showed a trend to reduce ~150 Kcal of total energy daily intake and a decrease in fat consumption (*p* = 0.04), probably due to satiety induction through the fiber ingestion increase by MexMix (Table S10).

No participant reported any adverse effects because of MexMix consumption. Noteworthily, the participants did not modify their physical activity during the intervention, meaning that they were mostly sedentary. We found a mean reduction of 300 g in body weight, 400 g in fat mass, and a clear trend toward a decrease in ALT serum levels; however, none of the measured variables was statistically significant (Table 1). 

On the other hand, we determined the effect of MexMix on participants’ gut microbiota (Table S11). The analysis showed a tendency toward an increase in alpha diversity (Figure 6B), as differences in the beta diversity were not found (Figure 6C). Also, MexMix supplementation did not modify any bacterial phylum abundance (Figure S3); however, a tendency toward a decrease in Proteobacteria was evident (Figure 6D), along with an increase in Lachnospiraceae abundance (Figure 6E). Remarkably, LEfSe analysis showed that four SFCA-producing bacteria characterized the 6-week supplementation with MexMix (Figure 6F). 

Body image satisfaction refers to how a person feels about their mental image of the size, appearance, and shape of their body. Here, we found a significant decrease in body dissatisfaction after MexMix administration (Figure 6G). PWB refers to the feeling of self-fulfillment and the promotion of maximum potential development of people; in this study, MexMix showed an evident trend toward an increase in PWB (Figure 6H). 

Furthermore, a significant positive correlation was observed between PWB and *Bifidobacterium*, a probiotic genera (Figure 6I). In addition, some families enriched by MexMix, like Lachnospiraceae, Butyricicoccaceae, and Christensenellaceae, negatively correlated with fat mass, weight, and BMI (Figure 6I). 

## 4. Discussion

In our study, supplementation with a mixture of prehispanic foodstuffs (nopal, cocoa, and cricket) demonstrated a prebiotic effect in mice and overweight/obese patients. MexMix supplementation significantly decreased (24%) the animal weight compared to the HF group. Previously, in a mild obesity model, we demonstrated that MexMix supplementation equalized mice weight to the ND group in as little as two weeks [6]; nonetheless, the amount of fat in this MASLD model was twice as much. Also, the weight reduction was more significant than in studies where the foods were used separately; supplementation with 8% *Acheta domesticus* on a high-fat diet showed a 19% reduction in body weight [16]. Similarly, supplementation with 8% cocoa powder for ten weeks in obese mice reduced only 5% of the body weight [17].

Previously, supplementation with nopal (4%) or cacao (8%) failed to meaningly reduce the epididymal and visceral fat weight [17,18]. Therefore, supplementation with the MexMix mixture proposed here proved more effective in reducing epididymal and visceral fat by a greater percentage. In addition, here, we found a similar decrease in the TG, leptin, IR, insulin, and PAI-1 serum levels comparable to our previous mild obesity model [6], demonstrating that even though the mice used here had severe obesity, they underwent beneficial effects.

MexMix supplementation showed prebiotic effects by enriching 10 bacterial taxa, like Lachnospiraceae and Ruminococcaceae, which promote health and improve resistance to diseases, through the production of SCFAs, mainly acetate and butyrate [19,20]. Also, our data showed a negative correlation of both families with colon *Il6* expression, correlating with the study where patients with alcohol-induced cirrhosis showed a significant reduction in the IL6 plasma levels [21] when they received a fecal transplant enriched with Lachnospiraceae and Ruminococcaceae.

MexMix supplementation also enriched *Akkermansia*, a mucin-degrading probiotic [22]. Everard et al. demonstrated that the *Akkermansia muciniphila* levels are reduced in obese and T2DM mice, but administering it restored their metabolic profile and increased their endocannabinoids, regulating inflammation and intestinal functions [23]. Therefore, it could be a keystone bacterium in the supplemented group as it exhibited a significant positive correlation with the insulin-sensitizing adipokine, adiponectin.

MexMix increased the *Eubacterium_coprostanoligenes_group* abundance. Li et al. found that *Eubacterium coprostanoligenes* lowered the plasma cholesterol in germ-free mice [24]. Our MexMix results in MASLD demonstrated a notable prebiotic effect, exceeding prior studies with isolated components of our foodstuff that increased a maximum of five bacterial taxa [25,26]. 

Intestinal health restoration by MexMix supplementation could be mediated by SCFA production, mainly acetate and propionate. It has been shown that acetate stimulates ZO-1 and occludin protein expression in CaCo-2 cells [27]. In addition, SCFAs can directly activate G-coupled receptors (GPRs); GPR43 activation is essential for intestinal homeostasis [28] and acetate and propionate are the most potent activators of GPR43 [29]. Previously, 5% nopal dietary fiber increased the expression of GPR43 in rats [30] and supplementation with 10% cocoa in ZDF rats (Zucker Diabetic Fatty Rat) increased the acetate levels higher than the healthy group and restored propionate levels and showed no differences in the butyrate levels, which resulted in an increase of the crypt size, expression of ZO-1 and a decrease in IL6 and TNF-α levels; results similar to ours [25].

Moreover, another significant result of this study is the upregulation of oxidoreductase expression, the substantial increase in NRF2 nuclear translocation and, consequently, the higher expression of *Sod* and *Cat*, even more than in the ND group. It has been shown that polyphenols can activate NRF2, which induces various antioxidant enzymes, resulting in reduced oxidative stress [31,32]. In addition, supplementation with 10% cocoa powder on a high-fat diet significantly increased the *Sod* and *Cat* mRNA levels; however, it showed similar levels to their ND group [33]. In our study, the elevated levels, likely attributed to combined nopal and cocoa polyphenols, accentuate the superiority of the three combined nutraceuticals for supplementation over individual ones.

Interestingly, we demonstrated that MexMix increased H3-K14 acetylation. Although there is no information on the possible role of H3-K14 in MASLD, the increase in H3-K14 acetylation is associated with gene transcriptional activation [34]. Contrasting with these data, in rats with isoniazid-induced liver injury, H3-K14 expression was decreased compared to the undamaged group [35]. 

Moreover, MexMix downregulated miRNA-34a, miRNA-103, and miRNA-33a hepatic expression. To the best of our knowledge, there is no study prior to ours that evaluated the effect of nopal, cocoa, or cricket on the expression of hepatic miRNAs; thus, this work provides novel information on the epigenetic mechanisms by which these foods may be affecting gene expression. Several studies have shown that polyphenols can contribute to MASLD improvement through the expression regulation of various miRNAs; however, the mechanisms are still unknown [36]. Therefore, it is plausible to affirm that the beneficial effects of the MexMix diet on insulin resistance and lipid metabolism in the liver could be modulated by changes in the expression of miRNAs, since MexMix has a high number and concentration of polyphenols. Moreover, changes in miRNA expression could also be modulated by changes in intestinal microbiota, since we found multiple significant correlations between bacterial taxa and miRNA expression (Figure 5D). The microbiota’s influence on miRNA expression is an emerging field and there is little information about it [15,37,38]. Thus, our results provide relevant information for this field, specifically concerning MASLD and hepatic miRNAs.

Developing user-friendly strategies for weight reduction is crucial for treating obesity/MASLD. Our study confirms MexMix’s tolerability and safety in overweight/obese individuals. While the pilot study did not show significant changes in parameters, potential limitations include the uncontrolled calorie intake and short consumption duration. Thus, a randomized placebo-controlled trial with a restrictive diet and longer MexMix consumption is essential for a comprehensive assessment.

Despite this, we demonstrated a prebiotic effect of MexMix in human participants. Correlating with the results in mice, two members of Lachnospiraceae were found to be increased, *Agathobacter* and *Eubacterium_eligens_group*. *Agathobacter* is mainly a butyrate and acetate producer [39]. Previous supplementation with bread rich in barley beta glucans [40] and a diet of high fiber rye food [41] were associated with increased *Agathobacter*. Christensenellaceae enrichment, observed following MexMix supplementation, has been associated with a reduction in visceral adipose tissue [42] and a lower BMI [43]. The negative correlation between Christensenellaceae and VFA and body weight found in this study suggests that this family may play a role in mediating the observed tendencies toward decreases in VFA and weight.

Finally, body image dissatisfaction is a prominent contributing factor in the development of eating disorders [44]. Consequently, interventions that aim to reduce weight and improve general health should consider addressing this psychopathological attribute. Thus, it is important to point out that our study provides evidence of the efficacy of MexMix supplementation in reducing distress linked to body dissatisfaction.

In obese patients, one month of cooked nopal (300 g/day) showed increased Prevotella enrichment [45]. Cricket powder (25 g/day for 14 days) in healthy adults boosted *Bifidobacterium animalis* [46]. Similarly, cocoa flavanol (494 mg/day for one month) increased *Bifidobacterium* and *Lactobacillus* in healthy individuals [47]. These findings corroborate in humans that a combination of the three nutraceuticals demonstrated a superior prebiotic effect to the individual components. 

## 5. Conclusions

MexMix supplementation exhibited multiple beneficial effects due to the blending of three nutraceuticals, which were not observed when tested in isolation. This finding adds meaningful nutritious and social value to foods that are part of the ancestral prehispanic cuisine. Moreover, we have elucidated the underlying mechanisms through which this dietary intervention functions, providing sufficient evidence for the potential therapeutic application of MexMix in diseases associated with high-fat and high-sugar diets.

## Figures and Tables

**Table 1:** Anthropometric and biochemical variables of participants before and after MexMix supplementation.

	Baseline	6-Week	*p*
Weight (kg)	82.7 ± 9.77	82.34 ± 9.78	0.227
BMI (kg/m^2^)	30.42 ± 3.49	30.31 ± 3.59	0.318
VFA (cm^2^)	122.59 ± 28.85	123.58± 31.19	0.480
Muscle Mass (kg)	27.41± 4.97	27.52± 4.97	0.478
Fat Mass (kg)	33.28 ± 9.57	32.91 ± 9.71	0.155
Body Fat (%)	39.78 ± 9.19	39.21 ± 9.78	0.295
Obesity Degree (%)	140 ± 16.75	139.44 ± 17.39	0.270
Cholesterol (mg/dL)	182.44 ± 36.41	199.19 ± 30.78	0.081
Triglycerides (mg/dL)	130.75 ± 58.62	126.12 ± 53.94	0.594
HDL (mg/dL)	51.13 ± 12.41	53.06 ± 11.33	0.374
LDL (mg/dL)	113.6 ± 26.25	120.03 ± 19.35	0.272
VLDL (mg/dL)	26.06 ± 11.68	25.23 ± 10.74	0.627
CHOL/dHLD	3.67 ± 0.84	3.89 ± 0.85	0.061
AST (U/L)	26.75 ± 7.79	26.43 ± 5.29	0.846
ALT (U/L)	24.13 ± 13.19	20.69 ± 9.18	0.064

## Data Availability

Data are contained within the article.

## Supplementary Materials

The following supporting information can be downloaded at: https://www.mdpi.com/article/10.3390/antiox13060664/s1, Table S1: Diet and drink water content in mice groups; Table S2: Catalog number of antibodies used to evaluate protein expression; Table S3: Primers sequence for gene 16S rRNA amplification; Table S4: Catalog number of specific probes to evaluate miRNA and gene expression; Table S5: Composition of MexMix powder daily dose; Table S6: Mean dietary intake in mice during experimental phases; Table S7: Sequences summary of mice model; Table S8: Relative abundance of phyla among mice groups; Table S9: Kyoto Encyclopedia of Genes and Genomes (KEGG) pathways with significantly differential abundance between MexMix and HF/FS groups; Table S10: Total daily intake before and after MexMix intervention; Table S11: Sequences summary of pilot study; Figure S1: Serum levels of Ghrelin, Glucagon, GLP-1, GIP and Resistin in MAFLD mice model; Figure S2: Heatmap of Spearman correlations between taxa and variables associated with intestinal and metabolic health in mice; Figure S3: Pie chart of most abundant bacterial phyla.
