# Supplementary material for: A Novel Foodstuff Mixture Improves the Gut–Liver Axis in MASLD Mice and the Gut Microbiota in Overweight/Obese Patients
Source: Antioxidants (Basel). 2024 May 29;13(6):664. doi: 10.3390/antiox13060664 (PMC11200377; doi:10.3390/antiox13060664)
Supplement: Supplementary file 1 [file antioxidants-13-00664-s001.zip › antioxidants-2986594-supplementary.pdf]

Table S1: Diet and drink water content in mice groups

	ND		HF/FS		MexMix	
	**g/100gr**	**% Kcal**	**g/100gr**	**% Kcal**	**g/100gr**	**% Kcal**
**Proteins**	18.6	24	16.73	13.18	18.70	14.80
**Carbohydrates**	44.2	58	33.79	26.61	30.94	24.48
**Fat**	6.2	18	33.98	60.21	34.11	60.72
**Nopal**	-	-	-	-	6.78	-
**Cricket**	-	-	-	-	8.47	-
**Cocoa**	-	-	-	-	8.47	-
**Total fiber**	3.5	-	4.26	-	8.53	-
**Kcal/gr**	3.1		5.08		5.06	
**Water (2.31% fructose, 1.89 % sucrose)**	No		Yes		Yes	

Table S2: Catalog number of antibodies used to evaluate protein expression.

Protein	Catalog number
NFR2	ab92946
H3K14	sc-518011
Lamin-B1	sc-374015
CD45	ab281586
Claudin-1	sc-166338

Table S3: Primers sequence for gene 16S rRNA amplification.

16S gene	Sequence
Forward	5´TCGTCGGCAGCGTCAGATGTGTATAAGAGACAGCCTACGGGNGGCWGCAG-3´
Reverse	5´GTCTCGTGGGCTCGGAGATGTGTATAAGAGACAGGACTACHVGGGTATCTAATCC-3´

Table S4: Catalog number of specific probes to evaluate miRNA and gene expression.

miRNA	Catalog number	Gen	Catalog number
mmu-miR-34a-5p	mmu481304_mir	*Tnfa*	Mm00443258_m1
mmu-miR-33	Mm04238236_s1	*Il6*	Mm00446190_m1
mmu-miR-122-5p	mmu480899_mir	*Ocln*	Mm00500912_m1
mmu-miR-103-3p	mmu478253_mir	*Cldn1*	Mm01145393_m1
mmu-miR-16-5p	mmu482960_mir	*Tlr4*	Mm00445273_m1
		Myd88	Mm00440338_m1
		Ppara	Mm00440939_m1
		Pparg	Mm00440940_m1
		Sod	Mm01344233_g1
		Cat	Mm00437992_m1
		Gapdh	Mm99999915_g1

Tnfa = tumor Necrosis Factor alpha, *Il6* = interleukin 6, *Ocln*= Occludin, *Cldn1*= Claudin 1, *Tlr4*= Toll-like receptor 4, *Myd88*= Myeloid differentiation primary response protein, *Ppara*= Peroxisome proliferator activated receptor alpha, *Pparg*= Peroxisome proliferator activated receptor gamma, *Sod*= Superoxide Dismutase, *Cat*=Catalase and *Gapdh*= glyceraldehyde-3-phosphate dehydrogenase.

Table S5: Composition of MexMix powder daily dose.

		Cocoa (10g)	Crickets (10g)	Nopal (10g)	MexMix Total (30g)
Proteins	g	2	6.5	0.5	9
Total fat	g	1.4	1.6	0	3
Saturated fat	g	0.8	0.6	0	1.4
Carbohydrates	g	0.8	2.5	2	5.3
Sugars	g	0.2	0	0	0.2
Fiber	g	3.3	1.9	4.5	9.7
Energy	Kcal	23.2	50.4	45.4	119

Table S6: Mean dietary intake in mice during experimental phases.

		ND	HF/FS	MexMix
**Prior treatment**				
Daily energy intake	Kcal	10.59 ± 0.67[a]	13.85 ± 2.35[b]	12.15 ± 2.22[a,b]
Daily food intake	g	3.42 ± 0.22[a]	2.82 ± 0.48[b]	2.47 ± 0.44[b]
Diet fat percentage	%	18	60.21	60.21
Daily fat intake	Kcal	1.91 ± 0.12[a]	8.34 ± 1.42[b]	7.32 ± 1.32[b]
**During treatment**				
Daily energy intake	Kcal	10.88±1.10[a]	12.13 ± 1.28[b; *]	14.62 ± 1.91[c; **]
Daily food intake	g	3.51 ± 0.35[a]	2.387 ± 0.25[b; *]	2.89 ± 0.38[c; **]
Diet fat percentage	%	18	60.21	60.72
Daily fat intake	Kcal	1.96 ± 0.20[a]	7.73 ± 0.77[b; *]	8.88 ± 1.16[c; **]

Values represent mean ± SD.
a, b, c: Differences between groups, ANOVA *post hoc* Tukey.
*, **, ***: Prior treatment versus during treatment, T-student paired.

Table S7: Sequences summary (n=24) of mice model.

Parameter	Fecal samples n=24
Total of raw read sequences	3,032,219
mean	144,391.380952
min-max	76,596 –182,696
Number of features [1]	2,150
Feature counts	1,007,090
count mean	49,026.0
count min–max	30,001-62,728

[1]Summary of sequences after trimming at 220 nt and denoized with dada2.

Table S8: Relative abundance of phyla among mice groups.

Phylum	ND	HF/FS	MexMix	p-value
Firmicutes	48.28 ± 15.43	28.02 ± 7.75	62.43 ± 6.94	**0.006**
Bacteroidetes	39.59 ± 15.27	64.33 ±11.16	24.98 ± 4. 21	**0.007**
Campilobacterota	4.01 ± 3.31	2.86 ± 2.93	4.63 ± 3.11	0.221
Proteobacteria	4.74 ± 2.36	1.74 ± 0.33	2.04 ± 0.62	0.054
Cyanobacteria	2.00 ± 1.15	0.92 ± 0.50	2.56 ± 1.11	**0.038**
Desulfobacterota	0.69 ± 0.34	1.47 ± 1.19	0.83 ± 0.16	0.288
Verrucomicrobiota	0.15 ± 0.15	0.17 ± 0.13	1.29 ± 0.65	**0.012**
Otros	0.52 ± 0.31	0.47 ± 0.31	1.23 ± 0.57	0.051

Data are represented as mean ± standard deviation. p-values were calculated using Kruskal Wallis test.

Table S9: Kyoto Encyclopedia of Genes and Genomes (KEGG) pathways with significantly differential abundance between MexMix and HF/FS groups.

Feature	Pathway name	p adjust
ko05340	Primary immunodeficiency	**0.0166**
ko00562	Inositol phosphate metabolism	**0.0072**
ko00440	Phosphonate and phosphinate metabolism	**0.0020**
ko04940	Type I diabetes mellitus	**0.0451**
ko04146	Peroxisome	**0.0109**
ko00600	Sphingolipid metabolism	**0.0024**
ko04141	Protein processing in endoplasmic reticulum	**0.0441**
ko04142	Lysosome	**0.0020**
ko00604	Glycosphingolipid biosynthesis - ganglio series	**0.0053**
ko05142	Chagas disease	**0.0049**
ko00510	N-Glycan biosynthesis	**0.0013**
ko04974	Protein digestion and absorption	**0.0105**
ko00524	Neomycin, kanamycin and gentamicin biosynthesis	**0.0480**
ko00950	Isoquinoline alkaloid biosynthesis	**0.0276**
ko00592	alpha-Linolenic acid metabolism	**0.0013**
ko04964	Proximal tubule bicarbonate reclamation	**0.0053**
ko00624	Polycyclic aromatic hydrocarbon degradation	**0.0020**
ko00626	Naphthalene degradation	**0.0020**
ko00623	Toluene degradation	**0.0046**
ko00940	Phenylpropanoid biosynthesis	**0.0013**
ko01053	Biosynthesis of siderophore group nonribosomal peptides	**0.0046**
ko00531	Glycosaminoglycan degradation	**0.0020**
ko00121	Secondary bile acid biosynthesis	**0.0005**
ko00430	Taurine and hypotaurine metabolism	**0.0260**
ko00130	Ubiquinone and other terpenoid-quinone biosynthesis	**0.0092**
ko00603	Glycosphingolipid biosynthesis - globo and isoglobo series	**0.0020**
ko05143	African trypanosomiasis	**0.0071**
ko00120	Primary bile acid biosynthesis	**0.0005**
ko00980	Metabolism of xenobiotics by cytochrome P450	**0.0064**
ko05150	Staphylococcus aureus infection	**0.0410**
ko00790	Folate biosynthesis	**0.0362**
ko00020	Citrate cycle (TCA cycle)	**0.0372**
ko03320	PPAR signaling pathway	**0.0225**
ko00460	Cyanoamino acid metabolism	**0.0020**
ko00830	Retinol metabolism	**0.0005**
ko00780	Biotin metabolism	**0.0064**
ko00511	Other glycan degradation	**0.0030**
ko00540	Lipopolysaccharide biosynthesis	**0.0085**
ko00982	Drug metabolism - cytochrome P450	**0.0064**
ko00140	Steroid hormone biosynthesis	**0.0102**
ko05100	Bacterial invasion of epithelial cells	**0.0127**
ko00908	Zeatin biosynthesis	**0.0105**
ko04210	Apoptosis	**0.0053**
ko00944	Flavone and flavonol biosynthesis	**0.0013**
ko04920	Adipocytokine signaling pathway	**0.0102**

Table S10: Total daily intake before and after MexMix intervention calculated by 24-hour dietary recall.

		**Baseline**	**Week 6**	**p-value**
Total energy	Kcal	2083 ± 426.5	1898 ± 339.8	0.093
Proteins	g	91.60 ± 23.49	83.95 ± 20.47	0.151
Carbohydrates	g	251.1 ± 77.97	239.9 ± 74.64	0.621
Fat	g	79.24 ± 18.03	65.89 ± 19.61	**0.041**
Fiber	g	18.67 ± 5.74	25.57 ± 6.15	**<0.0001**

Data are represented as mean ± standard deviation. p-values were calculated using T-Student paired test.

Table S11: Sequences summary (n=32) of pilot study.

**Parameter**	**Fecal samples n=32**
Total of raw read sequences	4,574,275
mean	142,946.09375
min-max	83,352 –168,112
Number of features [1]	2,290
Feature counts	1,346,514
count mean	42,078.5625
count min–max	22,355-60,303

[1]Summary of sequences after trimming at 220 nt and denoized with dada2.

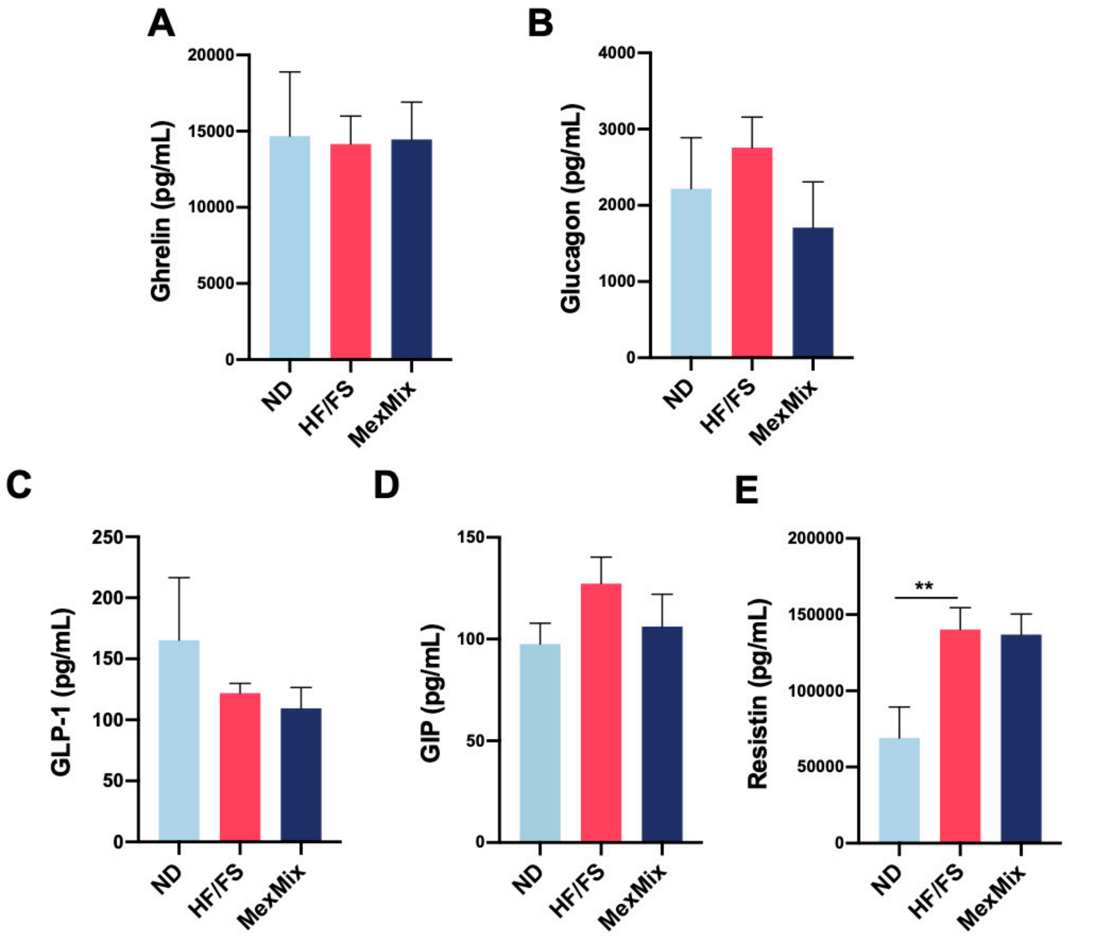

Figure S1: Serum levels of **(A)** Ghrelin, **(B)** Glucagon, **(C)** GLP-1, **(D)** GIP and **(E)** Resistin in MAFLD mice model.

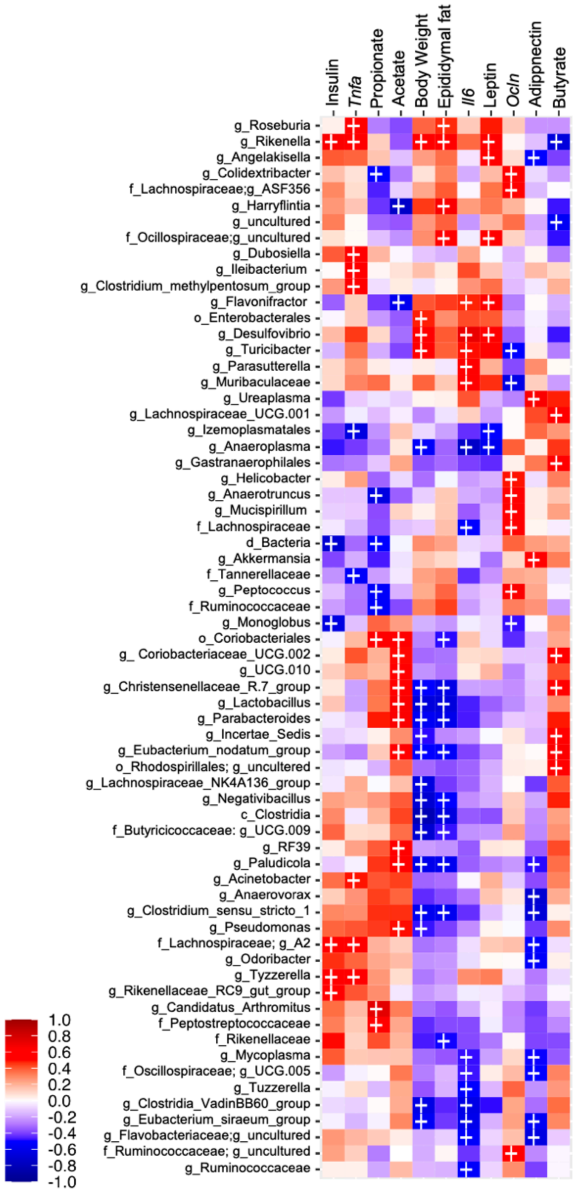

Figure S2: Heatmap of Spearman correlations between taxa and variables associated with intestinal and metabolic health in mice. Red shades indicate positive correlations, while blue shades indicate negative correlations. Statistically significant correlations ($p < 0.05$) are denoted by (+).

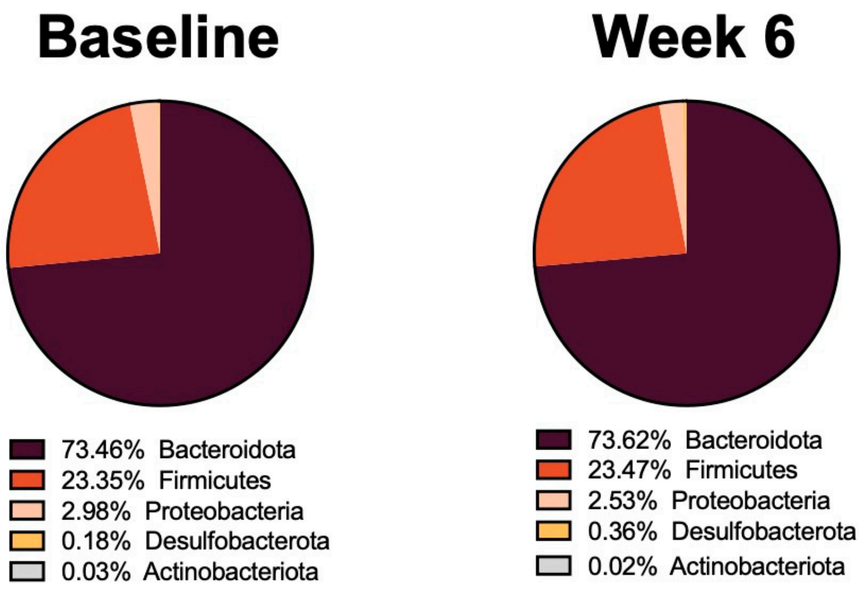

Figure S3: Pie chart of most abundant bacterial phyla (> 0.1%) and their relative abundance, before and after MexMix intervention on overweigth/obese participants.
